# Clinical Empathy, Personality Traits, and Resilience in Advanced Nursing Students: A Cross-Sectional Secondary Analysis

**DOI:** 10.3390/healthcare14111454

**Published:** 2026-05-25

**Authors:** Sonia Prieto de Benito, Ivan Herrera-Peco, Lina M. García-Nieto, Carlos Ruíz-Núñez, Andrés García-Notario, Silvia María Campos-Soler, Gema Mata-González, Fidel López-Espuela

**Affiliations:** 1 Hospital Clínico Universitario de Valladolid, Av. Ramón y Cajal, 3, 47003 Valladolid, Spain; sprietodb104@gmail.com; 2 University Center for Health Sciences-HM Hospitals (CUHMED), Camilo Jose Cela University, 28692 Madrid, Spain; gema.mata@ucjc.edu; 3 Instituto de Investigación Sanitaria HM Hospitales, 28015 Madrid, Spain; 4 Faculty of Biomedical and Health Sciences, Universidad Alfonso X El Sabio, 28691 Madrid, Spain; lgarcnie@uax.es (L.M.G.-N.); apsico@uax.es (A.G.-N.); scampso@uax.es (S.M.C.-S.); 5 Centro de Emergencias Sanitarias 061, Unidad de Innovación, 29590 Málaga, Spain; carlos.ruiz.nunez.sspa@juntadeandalucia.es; 6 Metabolic Bone Diseases Research Group, Nursing Department, Nursing and Occupational Therapy College, University of Extremadura, 10003 Caceres, Spain

**Keywords:** clinical empathy, nursing students, personality traits, resilience, person-centered care, ethical sensitivity

## Abstract

**Highlights:**

**What are the main findings?**

- In this exploratory sample of advanced nursing students, self-reported clinical empathy was associated with agreeableness and conscientiousness.
- Resilience, life satisfaction, academic engagement, and general self-efficacy were not significantly associated with total clinical empathy.
- In the exploratory multivariable cross-sectional model, agreeableness and conscientiousness were independently associated with total clinical empathy.

**What are the implications of the main findings?**

- The findings suggest that dispositional characteristics may be relevant correlates of self-reported clinical empathy, but they should not be interpreted as causal or predictive effects.
- Future longitudinal and multicenter studies should examine whether these associations are stable and whether they relate to observable educational or clinical outcomes.

**Abstract:**

**Background/Objectives:** Clinical empathy is a core competency in nursing education and is conceptually relevant to person-centered nursing care. However, limited evidence is available on how clinical empathy in advanced nursing students is associated with dispositional characteristics such as personality traits and resilience. This study aimed to examine cross-sectional associations between clinical empathy, Big Five personality traits, and resilience in third- and fourth-year nursing students. **Methods:** A descriptive, cross-sectional secondary analysis was conducted using an existing survey database. The final analytic sample comprised 66 third- and fourth-year nursing students from a nursing school in Spain. Clinical empathy was assessed with the Jefferson Scale of Empathy, resilience with the 6-item Brief Resilience Scale, and personality traits with the Big Five Inventory-44. Life satisfaction, academic engagement, and general self-efficacy were included as secondary psychosocial variables. Descriptive analyses, correlation analyses, group comparisons, and exploratory multiple linear regression were performed. **Results:** Higher agreeableness was associated with higher total clinical empathy (ρ = 0.390, *p* = 0.001) and perspective-taking (ρ = 0.440, *p* < 0.001). Higher conscientiousness was also associated with higher total clinical empathy (ρ = 0.480, *p* < 0.001), perspective-taking (ρ = 0.432, *p* < 0.001), and compassionate care (ρ = 0.324, *p* = 0.008). In the exploratory multivariable cross-sectional model, agreeableness and conscientiousness were independently associated with total clinical empathy, whereas resilience was not. Findings involving the Standing in the Patient’s Shoes subscale should be interpreted cautiously because of its low internal consistency. **Conclusions:** In this exploratory sample of advanced nursing students, self-reported clinical empathy was associated mainly with agreeableness and conscientiousness. These findings should be interpreted as cross-sectional associations based on self-report data and should not be taken as evidence of causal effects, ethical behavior, or person-centered care practices. Further longitudinal and multicenter studies are needed to examine whether these associations are stable and whether they relate to observable educational or clinical outcomes.

## 1. Introduction

Clinical empathy is a core competency in nursing education and practice and is widely recognized as an important component of high-quality health care [1,2,3]. In clinical settings, empathy should not be understood merely as a general affective disposition but rather as a professional capacity to understand the patient’s experience, consider the patient’s perspective, and respond appropriately to their needs, values, and circumstances [1,2]. In nursing students, this competency is particularly relevant because it develops during a formative period in which technical learning, clinical exposure, and professional socialization progressively converge. Previous studies have shown that empathy can be strengthened through educational strategies such as clinical simulation, reflective learning, and training focused on the therapeutic relationship [4,5,6]. The availability of validated instruments, including the Jefferson Scale of Empathy, has also facilitated the systematic assessment of empathy in health professions education [2].

Clinical empathy is also conceptually relevant to person-centered nursing care because it may support perspective-taking, therapeutic communication, and more attentive responses to patients’ experiences, needs, values, and circumstances [7,8,9,10]. From the perspective of the ethics of care, nursing practice involves not only technical competence but also attentiveness, responsiveness, and sensitivity to the person receiving care [7]. Similarly, person-centered care emphasizes that patients should not be reduced to their clinical condition but understood in relation to their preferences, values, and life context [10]. Empathy may contribute to this framework by supporting respectful, compassionate, and context-sensitive interactions [8,9]. However, empathy should not be equated with ethical behavior, moral sensitivity, person-centered care practices, moral deliberation, or clinical conduct. These constructs were not directly measured in the present study. Accordingly, the ethical and person-centered relevance of empathy is used here only as a conceptual background, whereas the empirical focus of this study is restricted to measured associations between clinical empathy, personality traits, and resilience.

The advanced stages of nursing education provide a relevant context for examining clinical empathy. During clinical training, students are progressively exposed to patient suffering, uncertainty, organizational constraints, and tensions between professional ideals and actual practice [11,12,13,14]. These experiences may foster empathy, moral sensitivity, and professional identity development, but they may also challenge students’ ability to sustain compassionate and person-centered responses in demanding environments [11,12,13]. Ethics education and reflective learning may support students’ moral sensitivity, although their effects depend not only on formal curricula but also on clinical learning environments, role modeling, and opportunities for reflection on practice [14]. Therefore, empathy in nursing students should be examined not as an isolated attribute but as a competency potentially associated with personal characteristics and educational context.

Personality traits represent one relevant set of personal characteristics. Personality describes relatively stable tendencies in how individuals perceive, regulate emotions, and relate to others. In care settings, these dispositions may influence how future professionals interpret patients’ needs, manage interpersonal closeness, and maintain appropriate responses in complex clinical situations. Evidence suggests that traits such as agreeableness, conscientiousness, and emotional stability are associated with prosocial relational styles, other-oriented behavior, and adaptive interpersonal functioning [15,16,17,18,19,20]. In nursing and related caring professions, previous studies have described associations between empathy and dimensions of the Five-Factor Model, as well as between personality and professional commitment among students in caring disciplines [18,19]. These traits should not be interpreted as direct measures of ethical behavior or clinical performance, but they may represent dispositional characteristics relevant to self-reported clinical empathy.

Resilience is another variable of interest in nursing education. Nursing students and professionals often face emotionally demanding situations, including exposure to suffering, academic and clinical pressure, and the need to maintain relational engagement under stress [21,22]. Resilience may help individuals adapt to adversity, recover from stress, and sustain professional functioning in challenging contexts [21]. Previous literature has also linked empathy with burnout, compassion fatigue, emotional exhaustion, and the quality of clinical engagement [23,24,25]. Although resilience may plausibly contribute to maintaining empathic responses in demanding situations, its direct association with clinical empathy in nursing students remains insufficiently established.

Although empathy in nursing education has been widely studied, much of the literature has focused on educational interventions to enhance empathy [4,5,6], its association with emotional intelligence and communication attitudes [26,27,28], or its relationship with moral sensitivity, caring behavior, and prosocial conduct [15,16,17,29,30]. These constructs are relevant but distinct from the dispositional variables examined in the present study. Personality traits may reflect relatively stable interpersonal and self-regulatory tendencies, whereas resilience reflects a perceived capacity to recover from stress. Previous work has examined empathy in relation to personality or professional commitment [18,19], and resilience has been studied as a relevant resource in nursing education and practice [21,22]. However, fewer studies have examined clinical empathy in advanced nursing students using an integrated dispositional framework that jointly considers Big Five personality traits and resilience [18,19,21,22]. Examining both sets of variables together may help clarify whether clinical empathy in advanced nursing students is more closely associated with interpersonal and self-regulatory dispositions, stress-adaptation resources, or both.

Therefore, this study aimed to examine cross-sectional associations between clinical empathy, Big Five personality traits, and resilience in advanced nursing students within a conceptual framework that considers empathy as a relational competency relevant to ethically sensitive, person-centered care.

## 2. Materials and Methods

### 2.1. Study Design

We conducted an observational, descriptive, cross-sectional study based on a secondary analysis of an existing survey database of undergraduate nursing students in Spain. The original database was generated from a broader survey designed to collect sociodemographic information and several psychosocial and dispositional self-report measures, including clinical empathy, resilience, personality traits, life satisfaction, academic engagement, and general self-efficacy. The reporting of this observational cross-sectional study was guided by the Strengthening the Reporting of Observational Studies in Epidemiology (STROBE) statement.

Because the present study was a secondary analysis, the data were not originally collected specifically to address the current research question. Therefore, the analysis was limited to the variables available in the original database, and the findings should be interpreted as exploratory cross-sectional associations rather than as evidence of causal, temporal, behavioral, or clinical effects.

For the present secondary analysis, the dataset was restricted to third- and fourth-year nursing students with complete data for the variables required to address the specific objective of this study. These students were considered to be in advanced stages of nursing education because they had greater exposure to clinical placements, patient contact, and professional role socialization than students in earlier academic years. Previous literature has shown that clinical learning environments play a central role in preparing nursing students for practice [31], while the final stages of training are especially relevant for the transition from student to registered nurse and for the consolidation of professional role expectations [32,33]. In addition, evidence from students with clinical practice experience suggests that professional socialization becomes particularly salient in third- and fourth-year nursing students, who are more directly involved in processes of adaptation to clinical settings and development of professional identity [34].

Accordingly, focusing on advanced nursing students provided a conceptually and methodologically coherent framework for examining cross-sectional associations between clinical empathy, personality traits, and resilience in students with more advanced clinical and professional socialization. This restriction also reduced conceptual heterogeneity by avoiding the combination of early-stage students with students who had already experienced more advanced clinical training.

### 2.2. Participants and Sample Size

The source population for the original survey consisted of undergraduate nursing students enrolled at the University of Extremadura nursing school in Spain. For the present secondary analysis, the final analytic sample consisted of 66 third- and fourth-year nursing students. These students were considered advanced nursing students because they were in later stages of undergraduate training and had greater expected exposure to clinical placements, patient contact, and professional role socialization than students in earlier academic years.

To be eligible for inclusion in the present analysis, participants had to (i) be enrolled in the undergraduate nursing program; (ii) be registered as third- or fourth-year students at the time of data collection; and (iii) have complete data for the key variables required for the present study, including clinical empathy, personality traits, resilience, age, and sex. Students from earlier academic years and records with incomplete data for the main study variables were excluded.

Because this was a secondary analysis of an existing survey database, the exact number of students who received the original invitation could not be retrieved from the dataset available for the present analysis. Therefore, the participation rate could not be calculated reliably. This limitation was considered when interpreting the findings, particularly in relation to potential selection bias.

The restriction to third- and fourth-year students was applied to reduce conceptual heterogeneity and to focus on students with more advanced clinical and professional socialization. However, this sampling decision does not eliminate the possibility of selection bias and should not be interpreted as producing a representative sample of nursing students. No a priori sample size calculation was performed specifically for the present study because the analysis was based on an existing dataset. The analytic sample was determined by the number of eligible students with complete data for the main variables. Given the modest sample size, the predominance of women in the sample, and the number of variables examined, all analyses were considered exploratory, particularly the multivariable regression model.

### 2.3. Data Collection and Instruments

Data were collected between October 2025 and November 2025 through an anonymous self-administered electronic questionnaire distributed via Microsoft Forms (Microsoft Corporation, Redmond, WA, USA), using an online format accessible from smartphones, computers, and tablets. The questionnaire was structured into two sections: a sociodemographic section and a second section containing the psychometric instruments used to assess the main study variables.

The sociodemographic section included sex, age, nationality, and study/work status. For the purposes of the present study, only records corresponding to third- and fourth-year nursing students with complete data for the main study variables were retained. Given the research aim, particular attention was paid to variables that could plausibly be associated with clinical empathy, particularly age and sex.

The second section included standardized self-report instruments assessing clinical empathy, resilience, personality traits, life satisfaction, academic engagement, and general self-efficacy. All instruments used in the present analysis were standardized self-report measures with prior evidence of validity and reliability in Spanish-speaking populations or in health sciences student samples. Internal consistency in the present sample was assessed using Cronbach’s alpha and is reported in Table 1. Because all study variables were collected through the same self-administered survey at a single time point, the findings should be interpreted as associations among self-reported measures and may be affected by common-method variance and social desirability bias.

Clinical empathy was assessed using the Jefferson Scale of Empathy, Health Professions Student version, a 20-item instrument widely used in health professions education to evaluate perspective-taking, compassionate care, and the ability to understand patients’ experiences in clinical settings [2]. Spanish versions of the Jefferson Scale of Empathy have shown adequate psychometric performance in health sciences students [35]. In the present sample, internal consistency for the total score was good. Because the Standing in the Patient’s Shoes subscale showed low internal consistency in the present sample, analyses involving this dimension were retained for descriptive completeness but interpreted cautiously and were not used as a basis for substantive conclusions.

Resilience was assessed using the Brief Resilience Scale, a 6-item instrument that evaluates the perceived ability to recover from stress and adapt to challenging situations [36]. Items are rated on a Likert-type response scale, with higher scores indicating greater resilience; negatively worded items were reverse-scored according to the original instrument instructions. This scale has shown evidence of reliability and validity in Spanish-speaking populations. In the present sample, internal consistency was acceptable.

Personality traits were assessed with the Big Five Inventory-44, which measures the five broad personality dimensions: extraversion, agreeableness, conscientiousness, neuroticism, and openness to experience. The Spanish version of the Big Five Inventory has demonstrated adequate psychometric properties in previous research [37]. In the present sample, internal consistency ranged from acceptable to good across the personality dimensions.

Life satisfaction was assessed with the Satisfaction with Life Scale, a 5-item measure of global cognitive well-being. The Spanish version has shown adequate psychometric properties in previous research [38]. Academic engagement was assessed with the Utrecht Work Engagement Scale for Students, 9-item version, which evaluates vigor, dedication, and absorption in academic activities and has shown adequate reliability in Spanish-speaking student samples [39]. General self-efficacy was assessed with the General Self-Efficacy Scale, which has shown evidence of validity and reliability in Spanish-speaking samples, including nursing students [40].

### 2.4. Ethical Issues

The study adhered to the Declaration of Helsinki and received approval from the Biosecurity and Bioethics Committee of Universidad de Extremadura [Ref. 1426]. Participation was anonymous and voluntary; respondents provided electronic informed consent and could withdraw at any stage without consequence. 

### 2.5. Statistical Analysis

Statistical analyses were performed using JAMOVI, version 2.7.24. Descriptive statistics were calculated for all study variables. Quantitative variables are summarized using means, standard deviations, medians, interquartile ranges, and 95% confidence intervals, while categorical variables are described using absolute and relative frequencies. The distribution of continuous variables was examined using the Shapiro–Wilk test. Internal consistency of the multi-item scales was assessed using Cronbach’s alpha.

Group comparisons by sex were examined using Student’s *t*-test for variables that approximated normality and the Mann–Whitney U test for variables that did not. Given the modest sample size and the imbalance between women and men, these comparisons were considered exploratory. Associations between age and the study variables, as well as between clinical empathy and dispositional or secondary psychosocial variables, were examined using Spearman’s rank-order correlation coefficient because several variables departed from normality and the study was exploratory in nature.

Given the number of bivariate analyses performed, no formal adjustment for multiple comparisons was applied. Therefore, *p* values from bivariate analyses should be interpreted cautiously, particularly those close to the conventional threshold of 0.05. Correlation results were interpreted primarily in terms of the consistency, direction, and magnitude of associations rather than isolated statistical significance.

To address the main study objective, an exploratory multiple linear regression model was estimated with total clinical empathy as the dependent variable. The model was specified a priori as a parsimonious, theory-informed model including the main dispositional variables of interest: agreeableness, conscientiousness, emotional stability, and resilience. Age and sex were included as adjustment variables because of their potential relevance to empathy in nursing education. Secondary psychosocial variables, including life satisfaction, academic engagement, and general self-efficacy, were examined in exploratory bivariate analyses but were not included in the regression model to avoid overfitting, given the modest sample size. Regression coefficients were interpreted as cross-sectional associations, not as evidence of causal, temporal, or predictive effects. Regression results are reported as unstandardized coefficients (B), standard errors, standardized coefficients (β), 95% confidence intervals, and *p* values. Collinearity diagnostics were examined using tolerance and the variance inflation factor (VIF). Statistical significance was set at *p* < 0.05, with the above cautions regarding exploratory analyses and multiple testing.

## 3. Results

### 3.1. Sample Description

The analytic sample included 66 third- and fourth-year nursing students, of whom 50 were women (75.8%) and 16 were men (24.2%). Age showed a slightly asymmetric distribution, with a mean of 22.6 years (SD = 3.0) and a median of 22.0 years [IQR: 21.0–23.0]. The sample was treated as a single analytic group to characterize its overall sociodemographic and psychometric profile before conducting exploratory inferential analyses (Table 1).

Regarding the main study variables, the total clinical empathy score had a mean of 113.62 (SD = 15.20) and a median of 117.00 [IQR: 107.25–124.00]. Among the empathy dimensions, Perspective-Taking and Compassionate Care showed the highest central tendency values. From a distributional standpoint, the total empathy score deviated from normality (Shapiro–Wilk W = 0.877; *p* < 0.001), as did Perspective-Taking (W = 0.817; *p* < 0.001) and Compassionate Care (W = 0.833; *p* < 0.001). In contrast, Standing in the Patient’s Shoes was the only empathy subscale that did not significantly depart from normality (W = 0.977; *p* = 0.260) (Table 1).

Resilience showed a mean of 27.95 (SD = 5.77) and a median of 28.50 [IQR: 24.00–31.00], with no evidence of departure from normality (W = 0.985; *p* = 0.607) (Table 1). For the personality traits, Extraversion (W = 0.975; *p* = 0.197), Conscientiousness (W = 0.979; *p* = 0.337), Neuroticism (W = 0.979; *p* = 0.311), Emotional Stability (W = 0.979; *p* = 0.311), and Openness (W = 0.987; *p* = 0.722) were approximately normally distributed, whereas Agreeableness showed a slight statistically significant deviation from normality (W = 0.963; *p* = 0.049). Among the secondary psychosocial variables, Life Satisfaction (W = 0.953; *p* = 0.014) and Academic Engagement (W = 0.901; *p* < 0.001) deviated from normality, while General Self-Efficacy did not significantly deviate from normality (W = 0.965; *p* = 0.058) (Table 1).

Internal consistency was acceptable to good for most multi-item scales, with Cronbach’s alpha values ranging from 0.700 to 0.899, except for the Standing in the Patient’s Shoes subscale, which showed low internal consistency (α = 0.439). Therefore, findings involving this subscale should be interpreted cautiously (Table 1).

### 3.2. Sex- and Age-Based Exploratory Comparisons

Sex-based exploratory comparisons are presented in Table A1. No statistically significant sex differences were observed for total clinical empathy or its subdimensions. Women showed a slightly higher median total empathy score than men (117.50 [IQR: 109.00–124.75] vs. 115.50 [IQR: 103.75–121.25]), but this difference was not statistically significant (U = 462.5, *p* = 0.353, r = 0.156). Similar nonsignificant patterns were observed for Perspective-Taking, Compassionate Care, and Standing in the Patient’s Shoes.

For the remaining study variables, most sex-based comparisons were not statistically significant. Openness to experience was higher among women than men (3.48 ± 0.60 vs. 3.16 ± 0.50; t = 1.96; *p* = 0.04; d = 0.56). However, this finding should be interpreted cautiously because the sex-based analyses were exploratory, the number of men was small, and no formal adjustment for multiple comparisons was applied.

Associations between age and the study variables are presented in Table 2. Age was not significantly associated with total clinical empathy or any empathy subdimension. Age was positively associated with agreeableness (ρ = 0.340, *p* = 0.005), indicating that older students tended to report higher agreeableness in this sample.

### 3.3. Association Between Clinical Empathy and Dispositional Variables

Associations between clinical empathy and the main dispositional variables are presented in Table 3. In this exploratory analysis, agreeableness and conscientiousness showed the most consistent pattern of associations with total clinical empathy and selected empathy dimensions. Other dispositional variables, including resilience, emotional stability, extraversion, and openness to experience, did not show statistically significant associations with total empathy. Because no formal adjustment for multiple comparisons was applied, results close to *p* = 0.05 should be interpreted cautiously.

Agreeableness was positively associated with total clinical empathy (ρ = 0.390; *p* = 0.001) and Perspective-Taking (ρ = 0.440; *p* < 0.001). Its association with Compassionate Care was positive but not statistically significant (ρ = 0.170; *p* = 0.173). Agreeableness also showed a near-threshold association with Standing in the Patient’s Shoes (ρ = 0.244; *p* = 0.049) (Table 3). However, this finding should be interpreted with caution because this subscale showed low internal consistency in the present sample, and no adjustment for multiple comparisons was applied.

Conscientiousness was positively associated with total clinical empathy (ρ = 0.480; *p* < 0.001), Perspective-Taking (ρ = 0.432; *p* < 0.001), and Compassionate Care (ρ = 0.324; *p* = 0.008). Its association with Standing in the Patient’s Shoes did not reach statistical significance (ρ = 0.218; *p* = 0.079). These findings indicate that, in this sample, conscientiousness was associated with several self-reported empathy outcomes, particularly total empathy and Perspective-Taking.

Resilience was not significantly associated with total clinical empathy (ρ = −0.054; *p* = 0.666) or with the empathy subdimensions. Emotional stability, extraversion, and openness to experience also did not show statistically significant associations with total empathy (Table 3). These null findings should be interpreted cautiously, given the modest sample size and exploratory nature of the analyses.

Exploratory correlations between empathy and secondary psychosocial variables are presented in Table A1. None of these variables showed statistically significant associations with total empathy or its subdimensions. For example, life satisfaction was not significantly related to total empathy (ρ = 0.191; *p* = 0.125), nor were academic engagement (ρ = 0.196; *p* = 0.115) or general self-efficacy (ρ = 0.085; *p* = 0.496) (Table A1).

### 3.4. Multivariable Model of Clinical Empathy

An exploratory multiple linear regression model was estimated with total clinical empathy as the dependent variable. The variables entered into the model were age, sex, agreeableness, conscientiousness, emotional stability, and resilience. As shown in Table 4, the overall model was statistically significant, F(6, 59) = 8.02, *p* < 0.001, with R^2^ = 0.449 and adjusted R^2^ = 0.393.

In this exploratory cross-sectional model, agreeableness was independently associated with total clinical empathy (B = 14.95, SE = 3.23, β = 0.531, 95% CI [8.487, 21.416], *p* < 0.001). Conscientiousness was also independently associated with total clinical empathy (B = 7.85, SE = 2.56, β = 0.326, 95% CI [2.733, 12.960], *p* = 0.003). These coefficients should be interpreted as cross-sectional associations, not as evidence of causal or temporal effects.

Age showed a negative association with total clinical empathy in the adjusted model (B = −0.89, SE = 0.43, β = −0.211, 95% CI [−1.750, −0.027], *p* = 0.043). However, this finding should be interpreted cautiously because age was not significantly associated with total empathy in the bivariate analysis, and the sample had a narrow age range. Sex, emotional stability, and resilience were not independently associated with total clinical empathy in the model.

Collinearity diagnostics did not indicate relevant multicollinearity problems. Tolerance values ranged from 0.709 to 0.919, and VIF values ranged from 1.089 to 1.410. Given the modest sample size and the exploratory nature of the model, regression estimates should be interpreted cautiously (Table 4).

## 4. Discussion

The present study found that, in this exploratory cross-sectional secondary analysis, self-reported clinical empathy was mainly associated with self-reported agreeableness and conscientiousness, whereas resilience and the secondary psychosocial variables were not significantly associated with total empathy. These findings should be interpreted as concurrent associations within a modest single-center sample and should not be understood as evidence of causal effects, temporal ordering, ethical behavior, person-centered care practices, or observed clinical performance. The results are consistent with previous literature that positions empathy as a relevant competency in health sciences education [1,2,3,4,5,6], but the present study does not allow conclusions about whether these associations translate into clinical conduct or care outcomes.

The associations observed for agreeableness and conscientiousness are consistent with prior studies linking dimensions of the Five-Factor Model to empathy-related variables and adaptive relational styles among professionals and students in caring disciplines [18,19,20]. Wan et al. [18] reported associations between Big Five traits and empathic behaviors in nurses, while Nesje [19] found that personality was related to professional commitment in nursing, social work, and teaching students. In the present study, agreeableness and conscientiousness were associated with total clinical empathy and selected empathy dimensions. However, these findings should not be interpreted as showing that these traits constitute a dispositional foundation or antecedent of empathic nursing practice. Because all variables were self-reported and collected through the same survey at a single time point, the observed associations may partly reflect shared method variance, socially desirable self-presentation, or conceptual overlap between self-rated empathy and socially valued personality traits.

The association between agreeableness and empathy is plausible because agreeableness reflects interpersonal orientation, cooperativeness, and concern for others, which may overlap conceptually with self-reported empathic attitudes. Similarly, conscientiousness may be related to self-regulatory tendencies that are compatible with attentive and responsible professional behavior. However, the present data do not permit conclusions about actual patient-focused behavior, clinical engagement, or ethical conduct. The findings should therefore be understood more narrowly: in this sample, students who reported higher agreeableness and conscientiousness also tended to report higher clinical empathy. This pattern may be relevant for understanding individual differences in empathy-related self-perceptions, but it requires replication using longitudinal designs, larger samples, and external assessments of clinical behavior.

The findings involving the “Standing in the Patient’s Shoes” subscale require caution. Although agreeableness showed a near-threshold association with this dimension, the subscale demonstrated low internal consistency in the present sample. Therefore, results involving this subscale should be considered unstable and secondary to findings based on the total empathy score and the more reliable dimensions of the Jefferson Scale of Empathy. This limitation is especially important because marginal associations may be sensitive to measurement error and to the absence of adjustment for multiple comparisons.

Resilience was not significantly associated with total clinical empathy or with the empathy subdimensions in either bivariate analyses or the exploratory multivariable model. This null finding should be interpreted descriptively and cautiously. Although resilience has been described as relevant to nurses’ emotional labor, adaptation to adversity, and protection against strain in demanding care contexts [21,23,24,25], the present study does not support a direct cross-sectional association between resilience and self-reported empathy in this sample of advanced nursing students. The data do not allow conclusions about whether resilience may have indirect, moderating, or context-dependent relationships with empathy; such possibilities would require specific longitudinal or mediation/moderation designs. Accordingly, the absence of significant associations should not be taken as evidence that resilience is unimportant in nursing education, but only that it was not directly associated with clinical empathy in the present analysis.

The absence of significant associations between clinical empathy and secondary psychosocial variables, including life satisfaction, academic engagement, and general self-efficacy, should also be interpreted cautiously. These variables may remain educationally relevant, but they did not show significant cross-sectional associations with clinical empathy in this sample. Given the modest sample size and the exploratory nature of the analyses, these null findings should not be interpreted as evidence of the absence of relationships in broader nursing student populations.

The exploratory analyses involving sex and age also require caution. Most sex-based comparisons were not statistically significant, but the small number of men limited the precision and interpretability of subgroup comparisons. Therefore, the absence of statistically significant sex differences should not be interpreted as evidence that such differences do not exist. Similarly, age was not significantly associated with total empathy in bivariate analyses but showed a small negative association in the adjusted model. This pattern may reflect model instability, suppression, or unmeasured differences among students rather than a substantive developmental association. It would therefore be inappropriate to conclude that empathy decreases with age.

The ethical and person-centered relevance of clinical empathy should be framed carefully. Empathy may support perspective-taking, therapeutic communication, and more attentive interactions with patients, which are conceptually relevant to person-centered nursing care and to the ethics of care [7,8,9,10]. However, the present study did not measure ethical sensitivity, moral deliberation, person-centered care behaviors, clinical performance, or patient outcomes. Therefore, the findings should not be interpreted as evidence that agreeableness or conscientiousness promotes ethically sensitive care. At most, the results identify self-reported dispositional correlates of clinical empathy, a construct that may be relevant to, but is not equivalent to, ethical or person-centered practice.

In relation to implications for clinical practice, nursing education, management, and research, the findings should be considered preliminary. At the educational level, the results suggest that empathy-oriented training may benefit from acknowledging that students differ in interpersonal and self-regulatory dispositions. However, personality-informed education should be used only to support reflection and professional development, not to label, rank, or select students. Educational strategies such as reflective learning, simulation, debriefing, mentoring, and guided discussion of clinical experiences may help students examine how their interpersonal tendencies shape their perceptions of patient care situations [11,12,13,14,31,32,33,34]. At the managerial and clinical education levels, supportive learning environments, constructive feedback, psychological safety, and positive role modeling remain important because students’ empathic development is shaped not only by individual characteristics but also by the clinical learning context [11,12,13,14,31,32,33,34]. At the research level, larger multicenter and longitudinal studies are needed to examine whether the associations observed here are stable over time and whether they relate to observed clinical behavior, supervisor-rated empathy, patient-reported experience, moral sensitivity, or person-centered care outcomes.

Nevertheless, this study has several limitations that should be considered when interpreting the findings. First, the cross-sectional design precludes causal or temporal inferences; therefore, the associations observed between personality traits and clinical empathy should not be interpreted as evidence that personality causes higher empathy. Second, the study was based on a modest sample from a single nursing school in Spain, which limits the generalizability of the findings to other educational institutions, countries, or curricular contexts. Third, the sample was predominantly female, reflecting the gender distribution commonly observed in nursing education but limiting the ability to examine sex-based differences with sufficient statistical balance. Fourth, because this was a secondary analysis of an existing survey database, the exact number of students who received the original invitation could not be retrieved, and the participation rate could not be calculated reliably; therefore, potential selection bias cannot be ruled out. Fifth, all variables were assessed using self-report instruments collected in the same survey, which may introduce social desirability bias, shared-method variance, and conceptual overlap among constructs. Sixth, some variables that could have strengthened the interpretation, such as direct measures of moral sensitivity, person-centered care behaviors, clinical performance, clinical placement characteristics, patient-reported outcomes, or supervisor-rated empathy, were not available. Seventh, no formal adjustment for multiple comparisons was applied, so marginal *p* values should be interpreted cautiously. Eighth, the ethical and person-centered dimensions of care were used only as a conceptual background, because ethically sensitive care and person-centered care were not directly measured; therefore, the findings should not be interpreted as evidence of ethical behavior or person-centered clinical practice. Finally, the Standing in the Patient’s Shoes subscale showed low internal consistency in this sample. Therefore, findings involving this dimension should be interpreted with caution and considered secondary to the results for the total empathy score and the more reliable empathy dimensions.

## 5. Conclusions

In this exploratory sample of advanced nursing students, self-reported clinical empathy was associated with agreeableness and conscientiousness, whereas resilience and broader psychosocial well-being variables were not significantly associated with total empathy. These findings should be interpreted as cautious cross-sectional associations within a modest single-center sample, not as evidence of causal, temporal, educational, ethical, or clinical effects.

The results suggest that agreeableness and conscientiousness may be relevant correlates of self-reported clinical empathy in this sample. However, because the study relied on self-report measures, did not directly assess ethical sensitivity, person-centered care behaviors, clinical performance, or patient outcomes, and did not use a longitudinal design, no conclusions can be drawn about whether these dispositional characteristics influence empathic development or translate into observable nursing practice.

Further multicenter and longitudinal studies using larger samples, external assessments, and direct measures of educational and clinical outcomes are needed to determine whether these associations are stable, replicable, and relevant to nursing education or practice.

## Figures and Tables

**Table 1:** Sociodemographic and psychometric characteristics of the third- and fourth-year nursing student sample.

Domain	Variable	Mean (SD)	Median [IQR]	Cronbach’s α
	Female, n (%)	—	50 (75.8)	—
	Male, n (%)	—	16 (24.2)	—
	Age (years)	22.6 (±3.0)	22.0 [21.0–23.0]	—
Clinical empathy	(JSE) Total	113.62 (15.20)	117.00 [107.25–124.00]	0.855
	Perspective-taking	60.38 (9.70)	64.00 [56.00–67.00]	0.897
	Compassionate care	44.50 (7.86)	47.50 [41.00–50.00]	0.791
	Standing in the patient’s shoes	8.74 (2.84)	9.00 [7.00–10.75]	0.439
Personality traits	Extraversion	3.26 (0.95)	3.31 [2.66–3.88]	0.891
	Agreeableness	3.92 (0.54)	4.00 [3.56–4.33]	0.736
	Conscientiousness	3.64 (0.63)	3.72 [3.11–4.11]	0.781
	Neuroticism	2.85 (0.66)	2.81 [2.50–3.22]	0.754
	Openness	3.40 (0.59)	3.40 [3.00–3.80]	0.777
	Emotional stability	3.15 (0.66)	3.19 [2.78–3.50]	0.7
Resilience	BRS total	27.95 (5.77)	28.50 [24.00–31.00]	0.73
Secondary psychosocial variables	Life satisfaction (SWLS)	24.85 (6.31)	26.00 [20.00–30.00]	0.854
	Academic engagement (UWES)	4.68 (1.20)	5.11 [3.83–5.64]	0.895
	General self-efficacy (GSE)	31.50 (4.96)	30.00 [28.25–34.75]	0.899

Note: SD = standard deviation; IQR = interquartile range; JSE = Jefferson Scale of Empathy; BRS = Brief Resilience Scale; SWLS = Satisfaction with Life Scale; UWES = Utrecht Work Engagement Scale; GSE = General Self-Efficacy Scale.

**Table 2:** Associations between age and study variables.

Variable		Association with Age
Clinical empathy total		ρ = −0.084; *p* = 0.503
	Perspective-taking	ρ = −0.008; *p* = 0.946
	Compassionate care	ρ = −0.159; *p* = 0.203
	Standing in the patient’s shoes	ρ = 0.015; *p* = 0.903
Personality Traits	Extraversion	ρ = 0.211; *p* = 0.089
	Agreeableness	ρ = 0.34; *p* = 0.005
	Conscientiousness	ρ = 0.049; *p* = 0.694
	Neuroticism	ρ = −0.074; *p* = 0.554
	Openness to experience	ρ = −0.036; *p* = 0.772
	Emotional stability	ρ = 0.074; *p* = 0.554
Resilience		ρ = 0.043; *p* = 0.73
Life satisfaction		ρ = −0.112; *p* = 0.37
Academic engagement		ρ = 0.183; *p* = 0.141
General self-efficacy		ρ = 0.113; *p* = 0.367

**Table 3:** Associations between clinical empathy and dispositional variables.

	Clinical Empathy Total	Perspective-Taking	Compassionate Care	Standing in the Patient’s Shoes
Extraversion	ρ = 0.117; *p* = 0.351	ρ = 0.214; *p* = 0.085	ρ = −0.009; *p* = 0.945	ρ = 0.054; *p* = 0.665
Agreeableness	ρ = 0.390; *p* = 0.001	ρ = 0.440; *p* = 0.001	ρ = 0.170; *p* = 0.173	ρ = 0.244; *p* = 0.049
Conscientiousness	ρ = 0.480; *p* = 0.001	ρ = 0.432; *p* = 0.001	ρ = 0.324; *p* = 0.008	ρ = 0.218; *p* = 0.079
Emotional stability	ρ = 0.018; *p* = 0.886	ρ = 0.046; *p* = 0.716	ρ = 0.052; *p* = 0.679	ρ = 0.017; *p* = 0.893
Openness to experience	ρ = 0.188 *p* = 0.131	ρ = 0.120; *p* = 0.338	ρ = 0.211; *p* = 0.089	ρ = 0.093; *p* = 0.457
Resilience (BRS total)	ρ = −0.054; *p* = 0.666	ρ = −0.028; *p* = 0.826	ρ = −0.110; *p* = 0.379	ρ = 0.015; *p* = 0.903

**Table 4:** The exploratory multiple linear regression model of total clinical empathy.

Predictor	B	SE B	β	95% CI for B	t	*p* Value	Tolerance	VIF
Age	−0.889	0.430	−0.211	[−1.750, −0.027]	−2.064	0.043	0.896	1.116
Sex (male)	−0.406	3.548	−0.012	[−7.505, 6.693]	−0.114	0.909	0.919	1.089
Agreeableness	14.952	3.231	0.531	[8.487, 21.416]	4.628	<0.001	0.709	1.410
Conscientiousness	7.846	2.555	0.326	[2.733, 12.960]	3.070	0.003	0.826	1.211
Emotional stability	−3.454	2.502	−0.149	[−8.461, 1.552]	−1.381	0.173	0.796	1.255
Resilience (BRS total)	−0.353	0.278	−0.134	[−0.910, 0.203]	−1.270	0.209	0.837	1.195

Note: The dependent variable was total clinical empathy (JSE total score). Sex was coded as male = 1 and female = 0. B = unstandardized coefficient; SE B = standard error of B; β = standardized coefficient; CI = confidence interval; VIF = variance inflation factor. Regression coefficients should be interpreted as cross-sectional associations.

## Data Availability

The datasets presented in this article are not readily available because the data are part of an ongoing study. In the meantime, additional materials and specific data supporting the findings of this study can be made available upon reasonable request to the corresponding author.

## Appendix A

**Table A1:** Exploratory associations between clinical empathy and secondary psychosocial variables.

	Clinical Empathy Total Rho (*p*)	Perspective-Taking Rho (*p*)	Compassionate Care Rho (*p*)	Standing in the Patient’s Shoes Rho (*p*)
Life satisfaction	ρ = 0.191; *p* = 0.125	ρ = 0.181; *p* = 0.146	ρ = 0.206; *p* = 0.098	ρ = 0.016; *p* = 0.897
Academic engagement	ρ = 0.196; *p* = 0.115	ρ = 0.194; *p* = 0.118	ρ = 0.057; *p* = 0.650	ρ = 0.204; *p* = 0.100
General self-efficacy	ρ = 0.085; *p* = 0.496	ρ = 0.118; *p* = 0.345	ρ = −0.002; *p* = 0.987	ρ = 0.119; *p* = 0.341
